# Supplementary material for: Comprehensive Annotation and Functional Exploration of MicroRNAs in Lettuce
Source: Front Plant Sci. 2021 Dec 24;12:781836. doi: 10.3389/fpls.2021.781836 (PMC8739914; doi:10.3389/fpls.2021.781836)
Supplement: Supplementary file 2 [file Data_Sheet_8.ZIP › GSM2230749.plot/Lsa-miRN533_Lsat_1_v5_gn_7_62580.1_1060_TPlot.pdf]

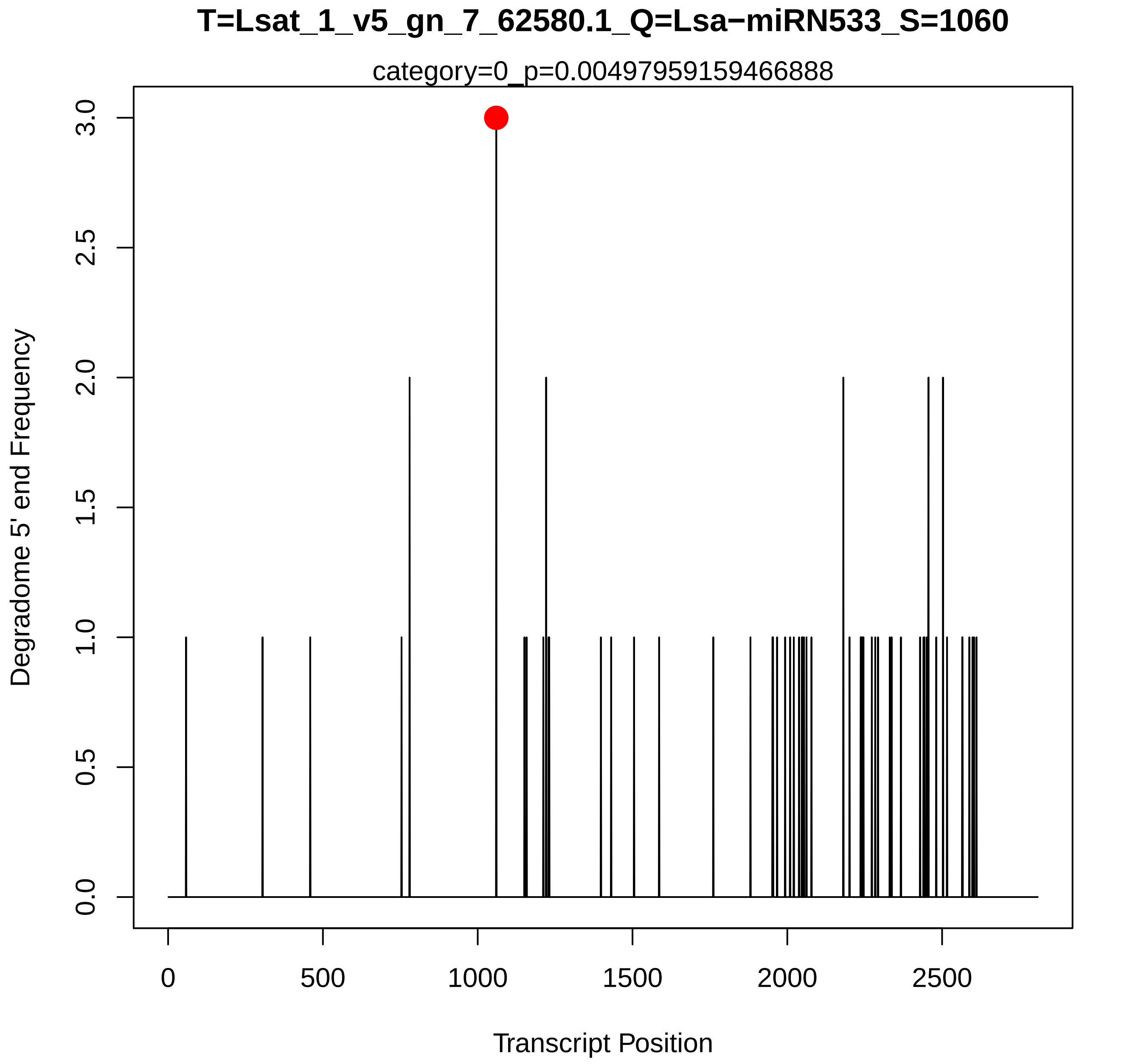
T=Lsat_1_v5_gn_7_62580.1_Q=Lsa−miRN533_S=1060
category=0_p=0.00497959159466888
Degradome 5' end Frequency
0.0
0.5
1.0
1.5
2.0
2.5
3.0
0
500
1000
1500
2000
2500
Transcript Position
